# Quality Reporting of Multivariable Regression Models in Observational Studies: Review of a Representative Sample of Articles Published in Biomedical Journals

**DOI:** 10.1097/MD.0000000000003653

**Published:** 2016-05-20

**Authors:** Jordi Real, Carles Forné, Albert Roso-Llorach, Jose M. Martínez-Sánchez

**Affiliations:** From the Unitat de Suport a la Recerca-Lleida, Institut Universitari d’Investigació en Atenció Primària Jordi Gol (IDIAP Jordi Gol), Barcelona (JR); Universitat International de Catalunya, Facultat de Medicina i Ciències de la Salut, Sant Cugat (JR, JMM-S); Department of Basic Medical Sciences, Universitat de Lleida, Lleida (CF); Oblikue Consulting (CF); Institut Universitari d’Investigació en Atenció Primària Jordi Gol, Barcelona (AR-L); and Tobacco Control Unit, Catalan Istitute of Oncology, Hospitalet de Llobregat (JMM-S), Spain.

## Abstract

Controlling for confounders is a crucial step in analytical observational studies, and multivariable models are widely used as statistical adjustment techniques. However, the validation of the assumptions of the multivariable regression models (MRMs) should be made clear in scientific reporting. The objective of this study is to review the quality of statistical reporting of the most commonly used MRMs (logistic, linear, and Cox regression) that were applied in analytical observational studies published between 2003 and 2014 by journals indexed in MEDLINE.

Review of a representative sample of articles indexed in MEDLINE (n = 428) with observational design and use of MRMs (logistic, linear, and Cox regression). We assessed the quality of reporting about: model assumptions and goodness-of-fit, interactions, sensitivity analysis, crude and adjusted effect estimate, and specification of more than 1 adjusted model.

The tests of underlying assumptions or goodness-of-fit of the MRMs used were described in 26.2% (95% CI: 22.0–30.3) of the articles and 18.5% (95% CI: 14.8–22.1) reported the interaction analysis. Reporting of all items assessed was higher in articles published in journals with a higher impact factor.

A low percentage of articles indexed in MEDLINE that used multivariable techniques provided information demonstrating rigorous application of the model selected as an adjustment method. Given the importance of these methods to the final results and conclusions of observational studies, greater rigor is required in reporting the use of MRMs in the scientific literature.

## Abstract 2

Supplemental Digital Content is available in the text

## INTRODUCTION

Two important aspects of biomedical research are the internal and external validity of the study design.^1^ Information bias and confounding variables affect internal validity and are present to some extent in all observational research. Information bias results from incorrect determination of the exposure, outcome, or both. Confounding is a “mixture” or “diffusion” of effects: a researcher attempts to associate an exposure with a result, but actually measures the effect of a third—sometimes unnoticed—factor, that is, a confounding variable. This bias can be diminished, but only if the confounding factor is anticipated and the relevant data are collected to allow proper adjustment.

Confounding factors can be controlled in various ways: restriction, matching, stratification, standardization, and multivariable techniques. All of these approaches are focused on achieving homogeneity between study groups,^1^ and in recent years multivariable regression models (MRMs) such as linear, logistic, Poisson, or Cox regression have become popular and very frequently used.^2^ A review of research based on Canada National Health Survey data found that nearly 80% of the studies used some type of MRM, predominantly logistical modeling.^3^ A systematic review of studies published by 10 prestigious journals in epidemiology and general medicine showed that almost 95% used MRMs, in addition to other techniques, as the adjustment methodology.^4^ The frequency with which any statistical method is applied is often determined by the available software and computational capacity; therefore, this high rate of MRM usage could be due to major advances in computational capabilities with increased availability of data, but also to the ease with which these techniques can now be applied using standard statistical software.^5^

An advantage of MRM analysis is that it allows the control of more confounding factors, compared to stratification, and a simultaneous evaluation of the relationship between several exposure factors and response variables of different types (continuous, dichotomous, count, or time-dependent events).^2,6^ The estimated effect of each variable reflects its association with the outcome, taking into account the contribution of the rest of the variables introduced into the model. However, modification effect is not identifiable by simple inclusion of the variable in the regression model; the interaction terms between exposure and effect modification, or the confounding variable, must also be included.

Moreover, MRMs assume probability distributions that include underlying assumptions (e.g., assumptions of normality, homoscedasticity, independence of errors, etc.). In addition, parameter estimation could be inefficient if there is multicollinearity between 2 or more variables, which affects convergence in the inference process, among other potential problems.^5,6^

Regression models produce nonbiased results for each variable of interest if the model is correctly specified and all potential confounding factors are included and correctly measured.^7^ Furthermore, if not all confounders are included or the model is not properly specified, the consequences are residual confounding and biased estimates.^8,9^ Although the underlying “true” model is seldom known, specification errors and residual confounding can be minimized by testing the formal assumptions of the selected model.^6,10^ Specific statistical tools are available to evaluate whether all necessary conditions have been met to apply a particular type of adjusted modeling and the appropriateness of the model that was finally selected.^6,10^ In addition, given that MRMs are usually sensitive to model specification, it is desirable to carry out more than 1 adjustment strategy to evaluate the stability of the estimated effects of different settings.^11^ All these measures, together with a sensitivity analysis (variation by subgroups) and interaction assessment, lead to more consistency in evaluating the adjusted measures of association and increase their validity and level of evidence.^12,13^

Various studies have described the statistical methodology used in published biomedical research.^3,4,14,15^ Strasak et al^15^ showed that inappropriate use of some statistical tests is one of the most common errors. In 2008, Groenwold et al^4^ carried out a systematic review of observational studies published in general medical and epidemiology journals with a high impact factor and reported finding poor quality in the adjustment methods used. More recently, in 2014, another systematic review of the use and application of generalized linear mixed models showed their increased use and, at the same time, room for improvement in reporting quality.^14^ However, there is a lack of evidence on the quality of reporting or the validation procedures used when MRMs are applied in observational studies. Therefore, the objective of the present study was to review the quality of statistical reporting when the most commonly used MRMs (logistic, linear, and Cox regression) were applied in analytical observational studies published between 2003 and 2014 by journals indexed in MEDLINE.

## MATERIALS AND METHODS

**FIGURE 1:**
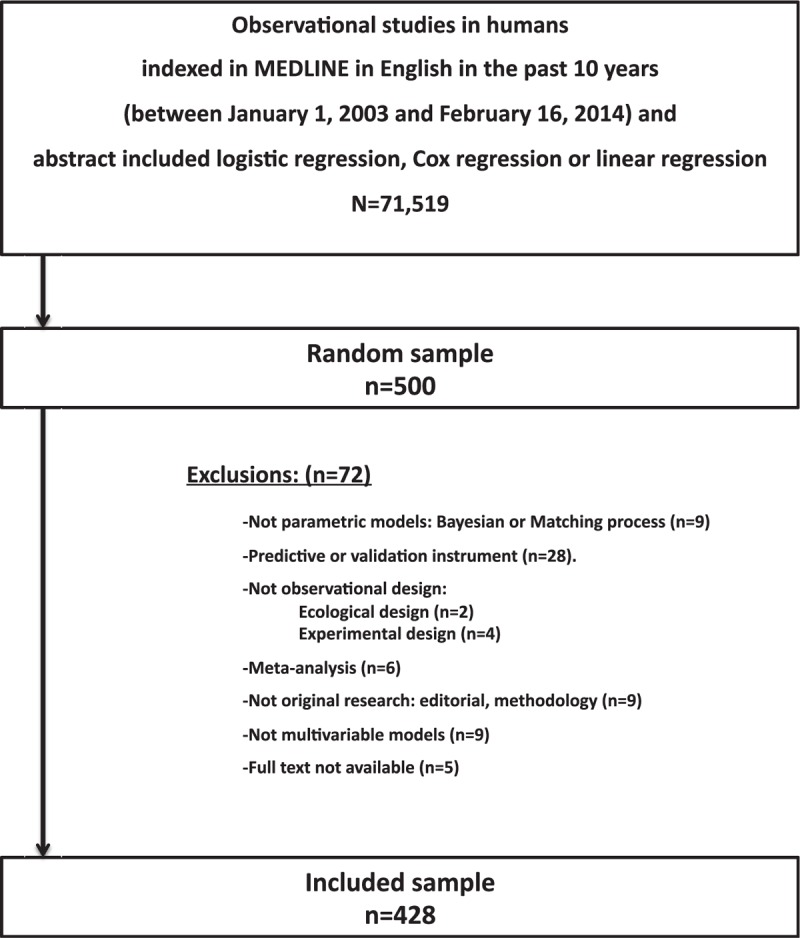
Flowchart of articles included.

We reviewed a representative random sample of articles indexed by MEDLINE using the PubMed search engine. The search was specifically designed to identify original studies with an analytical observational design that stated their use of logistic, Cox, or linear MRMs focused on confirmatory analysis (i.e., to assess the effect of exposure) (Supplementary Table S1 of the Appendix). The search was limited to studies in humans that were published in English between January 1, 2003 and February 16, 2014. Clinical trials, editorials, commentaries, and case reports were excluded. This strategy retrieved 71,519 references, from which a simple random sample of 500 articles was selected. A sample size of 500 randomly selected papers was calculated to allow estimation with 95% confidence and a precision of ±5% units, a population percentage considered to be of 50%. We assumed a 50% prevalence to maximize the sample size. A replacement rate of 20% was anticipated. Exclusion criteria removed 72 references, including those that proved to be focused on diagnosis, prognosis, or other analytical approaches. Therefore, 428 papers were finally reviewed (Figure 1).

### Items Reviewed in Full-Text Analysis

Based on the literature,^2,11,16,17^ a list of aspects related to the application of MRMs was specified, including testing formal assumptions, goodness of fit: interactions, and sensitivity analysis of the adjustment models (Table 1). An initial review of 10 articles served as a pilot test for the entire research team to define the list of items to be included, establish precise definitions, and improve interrater homogeneity. Finally, the definitive set of MRM-related items to be verified in each relevant section of the manuscript was established (Table 1). Each item was classified according to whether it would likely be stated in the methods section or was mainly involved in the communication of findings and would appear in the results section. If an item was reported in any section, the paper was considered to meet the criteria.

**TABLE 1:**
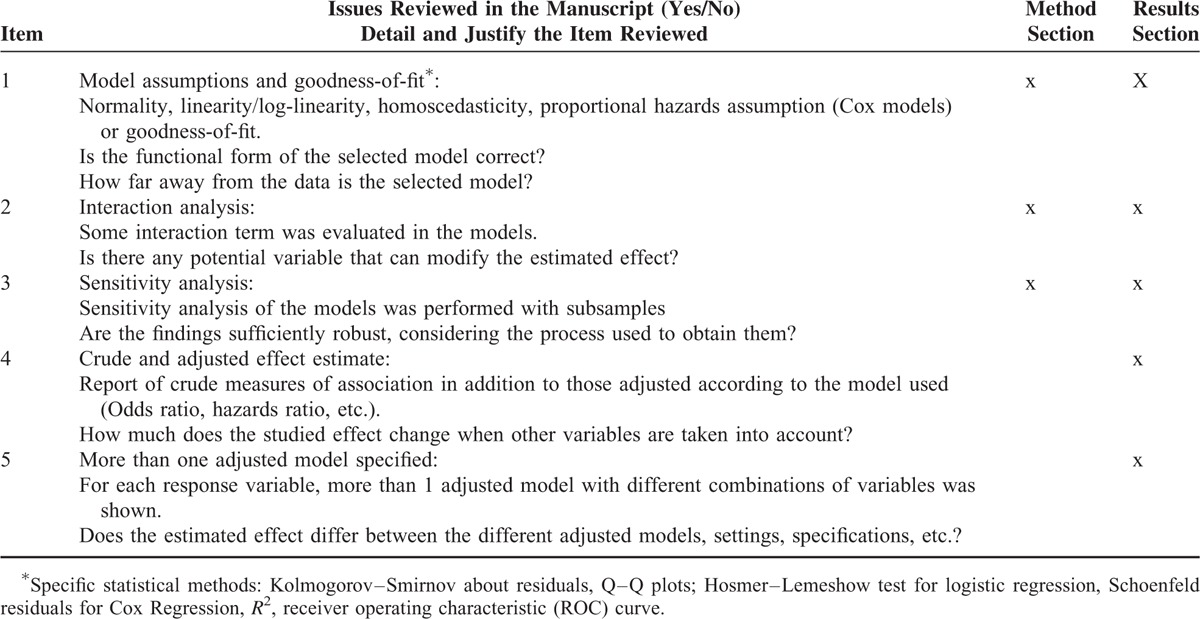
Primary Items Reviewed in Manuscripts of Observational Studies That Used Multivariable Methods (Logistic Regression, Cox Regression, or Linear Regression)

### Review Procedure

The selected articles were randomly distributed among the 3 designated reviewers on the research team. Any doubts were shared and resolved by consensus. In addition, 12 articles were randomly selected for reviewing by all 3 reviewers, for blinded evaluation of interrater agreement. No significant differences in outcomes between reviewers were observed (Supplementary Table S2); there was high interrater agreement (Kappa index > 0.73) and intraclass correlation coefficient of the number of completed items (0.88). The Kappa index measures the agreement between reviewers for compliance with each of the items separately (dummy variables) and the intraclass correlation coefficient quantifies the correlation of the number of completed items (numerical variable) between reviewers. A detailed analysis of intra- and interrater agreement is shown in the Appendix, Supplementary Table S2.

### Statistical Analysis

For each item specified for review, prevalence estimates and 95% confidence interval (CI) were obtained. We also calculated mean and standard deviation (SD) for the total number of review items fulfilled. CIs were computed using normal approximation. All analyses were stratified in groups according to the impact factor of the journal in the year of publication (≤2, 2–4, >4), sample size (<500, 500–1500, ≥1500), design (cross-sectional, cohort, and case–control), data source (ad hoc, clinical/administrative records, both, or “mixed”), and type of MRM (logistic, linear, and Cox). Pearson χ^2^ and trend tests were used to assess the association between prevalence of the items of interest and the categorical secondary variables. Mann–Whitney *U* test was used to examine the relationship between prevalence of items, sample size, and the journal's impact factor. We computed the 2-sided criteria for all variables and 1-sided criteria for the impact factor level because the higher the impact factor, greater rigor is required in reporting the use of MRMs in the scientific literature. To assess and control for possible interactions, the analysis was again stratified by impact factor, sample size, design, and type of modeling. Significance level was set at α = 0.05. All analysis was carried out using the SPSS statistical software, version 18.0. (PASW Statistics for Windows, Version 18.0. Chicago: SPSS Inc.).

Ethical statement: None required the approval of the Ethics Committee because the primary source was secondary data from published scientific articles.

## RESULTS

Of 428 articles reviewed, published in 313 journals (mean of impact factor = 3.38), 49.5% were cohort studies, with data primarily collected using questionnaires specifically designed to address the research objective (45.6%). The most frequently used type of modeling was logistic regression (67.5%), followed by Cox (22.9%) and linear (18%) regression. Nearly half (48.8%) of the articles reviewed were published during the last 3 years of our study period (2010–2013). Only 4% of the articles referenced any other publication that expanded on the methodology used in the study.

Table 2 shows the overall percentage observed for each of the items reviewed, and in relation to all selected variables. The major item that was reported most often (33.4%; 95% CI: 28.9–37.8%) was “crude and adjusted effect” (item 4, Table 2), followed by sensitivity analysis (32.7%; 95% CI: 28.3–37.1%). The least-reported item was interaction analysis (18.5%, 95% CI: 14.8%–22.1%). Testing the assumptions of the model and fitting more than 1 model were reported in 26.2% and 25.7% of the articles, respectively (items 1 and 5, Table 2).

**TABLE 2:**
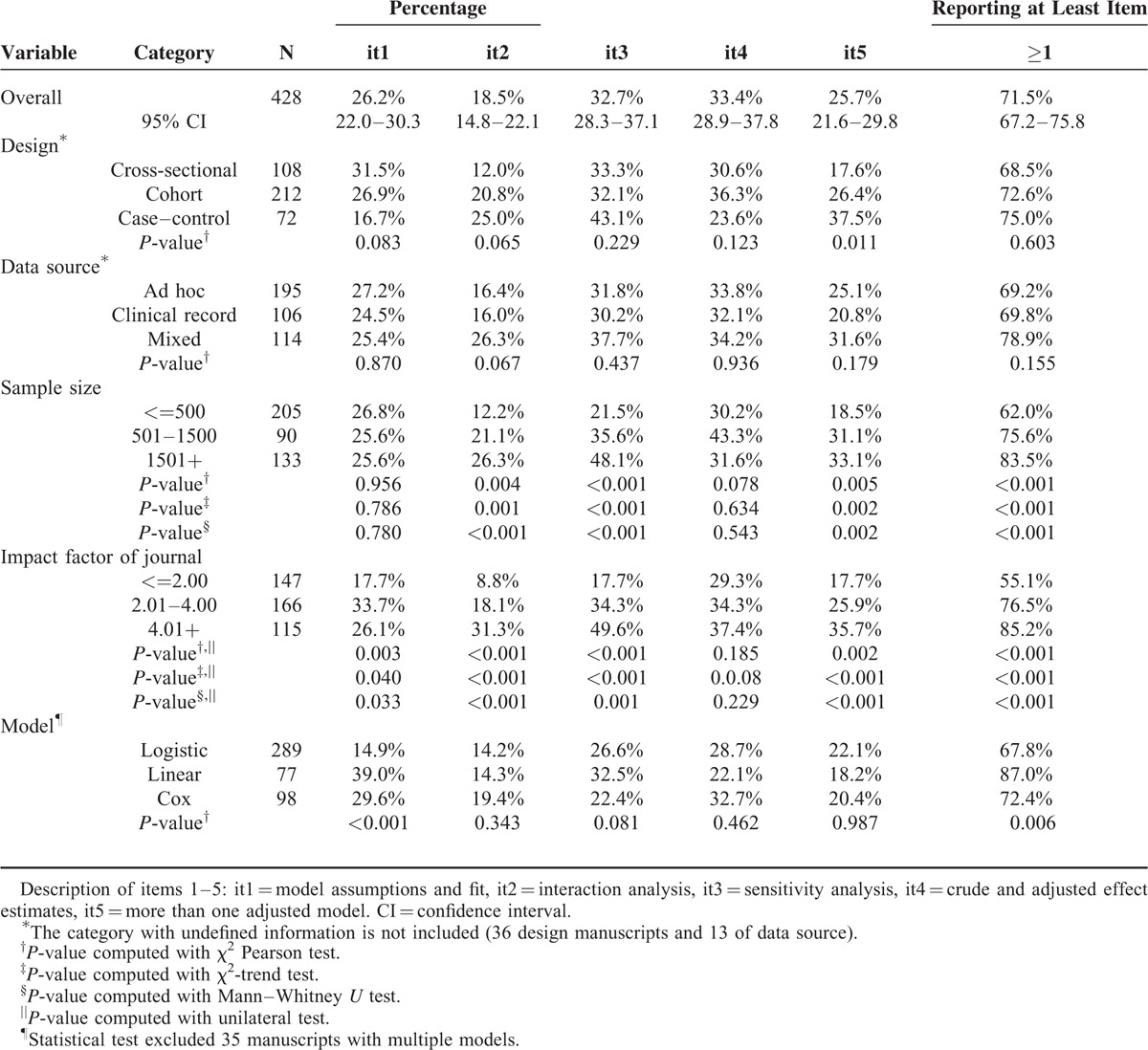
Frequency of Items Related to the Application of Statistical Models, Based on Study Characteristics in Articles Reviewed

The percentages observed for all of the items analyzed were higher in studies published in journals with a moderate or high impact factor (Table 2). The assessment of model adjustment criteria (item 1) was primarily observed in articles published in journals with a moderate impact factor and in studies that used linear models. The criteria referring to interactions, sensitivity analysis, and testing more than 1 model (items 2, 3, and 5, respectively) were also significantly and directly associated with sample size (Table 2).

**FIGURE 2:**
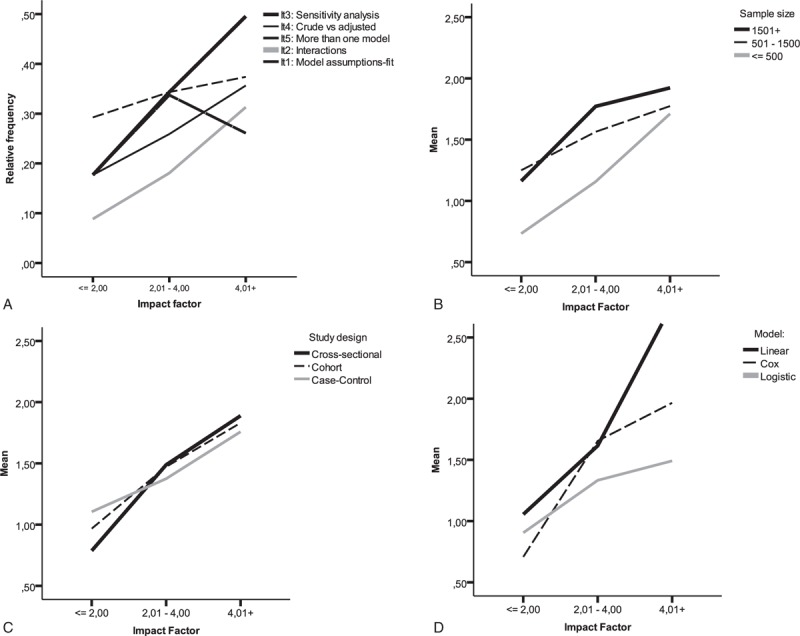
Relative frequency for each item searched (A), mean number of items per article (i.e., application of a multivariable regression model, stratified by impact factor and by sample size) (B), study design, (C) and type of model used (D).

The mean number of items identified in the articles reviewed was 1.36 (SD = 1.17), and increased with sample size and impact factor (*P* < 0.001). Both factors act independently of the mean number of items: there was no observed interaction between impact factor and sample size. Figure 2 shows how the frequency of each item increased with impact factor (Figure 2A), independently of sample size (Figure 2B), study design (Figure 2C), and type of MRM used (Figure 2D).

## DISCUSSION AND CONCLUSIONS

Our study shows very low reporting of MRM validation in observational studies indexed in MEDLINE, being higher in studies with larger sample sizes published in journals with a higher impact factor. Only 26.2% of the articles reviewed described their validation analysis of assumptions or goodness-of-fit for the MRM used, 33.4% showed both the crude and adjusted effects, and 32.7% described any sensitivity analysis. Interaction analysis was only observed in 18.5% of the articles reviewed.

Our results are consistent with previous scientific evidence.^4,14,18^ Müllner et al^18^ showed that journals with a higher impact factor had better statistical reporting, perhaps because their editorial process specifically includes statistical review. In our study, the percentages observed for all of the items analyzed were higher in studies published in journals with a higher impact factor. A systematic review by Casals et al^14^ of 108 articles that applied generalized linear mixed models, without discriminating between type of design or research objective, found that validation of the model and testing for goodness-of-fit were reported in 6.5% and 15.7%, respectively, of the articles. In contrast, our results showed a higher prevalence of this item (17.7%–33.7%, depending on the impact factor of the journal). This difference could be explained because our review is based on a random sample that included methodologies whose use is much more widespread.^19^ Another systematic review found a lack of attention to adjustment methods in analytical observational studies,^4^ in contrast with diagnostic, prognostic, or predictive validation studies in which combinations of variables were modeled with greater precision.^16,20–23^ In the latter types of studies, calibration, discriminatory power, goodness-of-fit, and validation of the statistical model are considered essential 1st steps before selecting the final adjusted model.^16^ The recent Transparent reporting of a multivariable prediction model for individual prognosis or diagnosis (TRIPOD) statement^24^ provides guidelines that highlight the essential aspects of developing and validating a predictive multivariable model. Requirements of this guide include, among others, the need for using internal validation methods to evaluate model's performance and to compare multiple models.^24^

On the other hand, published guidelines provide specific recommendations on the reporting of the scientific results of clinical trials (CONSORT),^25^ observational studies (STROBE),^26^ or statistical analysis in general scientific literature (SAMPL).^27^ These guidelines were developed to provide more complete and precise information about key aspects of research studies, and some have been incorporated into the author guidelines of major scientific journals. Nonetheless, even though the STROBE guidelines highlight the control of confounders as a crucial aspect of observational studies and the SAMPL and TRIPOD guidelines broaden the standards for the scrutiny of statistical methods, there is still a void in requiring or assessing multivariable methodology in observational designs. Notably, the Guide for Authors and Editors (Manual of Style for the American Medical Association) includes the need to report model diagnostics and proportion of variance explained by both individual variables and the complete model.^28^ In this sense, even though the data analysis may be correct, inadequate reporting makes it impossible for the reader to assess whether the data were processed appropriately.^18^

In observational research, best practice includes avoiding bias in the study design, adjusting for possible bias in the data analysis if it is not possible to avoid bias entirely in the design, and quantifying and analyzing the effects of residual bias on the study results.^7^ Nonetheless, if the model was not properly selected, there may be major residual confounding even after MRM adjustment,^29–31^ which leads to bias in the associations studied. For example, Liang et al^8^ recently published the results of a simulation study, concluding that “even when all confounding factors are known and controlled for using conventional multivariable analysis, the observed association between exposure and outcome can still be dominated by residual confounding effects.”

In this sense, overall goodness-of-fit, along with graphical validation analysis, can allow researchers to evaluate possible conflicts between the models and alert them to possible specification problems, even without ensuring that the model is completely correct.^32^ Properly fitting the model may require additional adjustment variables, their transformation, inclusion of interactions, or the choice of other adjustment techniques that are less sensitive to the selection of a particular model, such as stratified analysis, matching techniques,^33^ or other flexible modeling approaches.^34^

### Strengths and Limitations

One of the limitations of this analysis is that the primary source of data was the abstracts accessed in MEDLINE by the PubMed search engine. Therefore, the universe of potential studies for analysis was limited to that repository and the sensitivity of the search strategy used. In an effort to minimize missed records, we designed a highly specific search-term strategy. During our review we only had to discard 14.4% of the manuscripts for failing to meet at least 1 inclusion criterion.

Another limitation was that the quality or transparency of the methodological reporting could be affected by the word limitations imposed by a journal's guidelines. Nonetheless, it is now usually possible to complement an article with online supplementary information or to disseminate the methodological details and protocols in a separate manuscript that provides greater detail about the more technical aspects. However, only 4% of the manuscripts included in the present review contained any reference to a separate article detailing the methodology used.

We are aware that the items we reviewed need not have the same relevance and weight—and are not even always necessary. Assessing their interactions is not always justified, especially in small samples, and there are studies on medical interventions in which confounding could be considered negligible. Furthermore, there are other important aspects that would affect the quality of the analysis and results (e.g., the model was prespecified prior to undertaking the data analysis, or the research team had insufficient statistical background and knowledge).

Finally, our review was not paired and there could be a certain interrater variability. We attempted to minimize this potential limitation in 2 ways: very detailed specification of each item to be reviewed, all of which were easily identifiable; and prior training of reviewers with a pilot test. In addition, testing for agreement after completing the review showed a high level of intra- and interrater agreement (Supplementary Table S2).

## CONCLUSIONS

Statistical adjustment using MRM is a powerful tool for isolating the actual effect of exposure factors on potential confounders. However, the use of these techniques is not free of potential errors because they have strong underlying assumptions that must be tested. Our study showed that, despite the availability of known statistical tools that allow the evaluation of how well the models meet the conditions for their application, only a troublingly low percentage of published articles report information about model validation or measures to ensure the rigorous application of MRMs as an adjustment method. Given the importance of these statistical methods to the final conclusions, biomedical journals should require greater rigor in reporting the assumptions of the MRMs in the methods and results of observational studies.

## Supplementary Material

Supplemental Digital Content
