# Supplementary material for: Quality Reporting of Multivariable Regression Models in Observational Studies: Review of a Representative Sample of Articles Published in Biomedical Journals
Source: Medicine (Baltimore). 2016 May 20;95(20):e3653. doi: 10.1097/MD.0000000000003653 (PMC4902409; doi:10.1097/MD.0000000000003653)
Supplement: Supplemental Digital Content [file medi-95-e3653-s001.docx]

| TABLE S1. PubMed Search Strategy and List of Descriptors Used to Select Articles Published in English Between January 1, 2003 and February 16, 2014^*^ | | | | | | | | |
| --- | --- | --- | --- | --- | --- | --- | --- | --- |
| Search criteria in PubMed (n=71 519) | | | |  | Fields in PubMed | | | |
|  | Term or keyword used | | |  | MeSH | ptyp | tiab | text |
| Observational studies (n=1 150 728) | | |  |  |  |  |  |  |
|  | evaluation studies | | |  |  | + |  |  |
|  | evaluation studies as topic | | |  | + |  |  |  |
|  | evaluation study | |  |  |  |  | + |  |
|  | evaluation studies | |  |  |  |  | + |  |
|  | intervention studies | | |  | + |  |  |  |
|  | intervention study | |  |  |  |  | + |  |
|  | intervention studies | |  |  |  |  | + |  |
|  | case-control studies | | |  | + |  |  |  |
|  | case-control | |  |  |  | + |  |  |
|  | cohort studies | | |  | + |  |  |  |
|  | cohort |  |  |  |  |  | + |  |
|  | longitudinal studies | | |  | + |  |  |  |
|  | longitudinal | |  |  |  |  | + |  |
|  | longitudinally | |  |  |  |  | + |  |
|  | prospective | |  |  |  |  | + |  |
|  | prospectively | |  |  |  |  | + |  |
|  | retrospective studies | | |  | + |  |  |  |
|  | retrospective | |  |  |  |  | + |  |
|  | follow up | |  |  |  |  | + |  |
|  | comparative study | | |  |  | + | + |  |
|  | observational | |  |  |  |  | + |  |
| Human subjects | |  |  |  | + |  |  |  |
| Exclusion of clinical trials, editorials, commentaries or case reports (-) | | | | | | |  |  |
|  | editorial / letter / comment / case reports | |  |  |  | **-** |  |  |
|  | case report / case series | |  |  |  |  | **-** |  |
|  | clinical trial | | |  |  | **-** |  |  |
| Logistical model (n=61 739) | |  |  |  |  |  |  |  |
|  | logistic models | | |  |  |  |  | + |
|  | logistic regression | | |  |  |  |  | + |
|  | logistic regressions | | |  |  |  |  | + |
| Linear regression (n=22 965) | | |  |  |  |  |  |  |
|  | linear models | |  |  |  |  |  | + |
|  | linear regression | | |  |  |  |  | + |
|  | linear regressions | | |  |  |  |  | + |
| Cox regression model (n=28 853) | | |  |  |  |  |  |  |
|  | proportional hazards models | | |  |  |  |  | + |
|  | Cox regression | | |  |  |  |  | + |
|  | Cox regressions | | |  |  |  |  | + |
|  | Cox models | |  |  |  |  |  | + |
| MeSH= MeSH terms, ptyp= publication type, tiab= title or abstract, text= full-text access, += included term, -= excluded term,  *= search was executed on April 16, 2014. | | | | | | | | |
|  | | | | | | | | |

TABLE S2 (a). Inter-rater Agreement on the 12 Articles Evaluated in Common. Overall Agreement (%) and 2x2 Kappa

|  |  | Comparisons | | | | | |  |
| --- | --- | --- | --- | --- | --- | --- | --- | --- |
| Item description | | Reviewer 1 vs 2 | | Reviewer 1 vs 3 | | Reviewer 2 vs 3 | | Overall |
|  |  | % | Kappa | % | Kappa | % | Kappa | Agreement |
| Exclusions (3/12): 100% agreement | |  |  |  |  |  |  |  |
| 1.Model assumptions and goodness-of-fit | | 100.0% | 1.00 | 100.0% | 1.00 | 100.0% | 1.00 | 100% |
| 2.Interaction analysis: | | 100.0% | 1.00 | 100.0% | 1.00 | 100.0% | 1.00 | 100% |
| 3.Sensitivity analysis: | | 88.9% | 0.73 | 88.9% | 0.73 | 100.0% | 1.00 | 89% |
| 4.Crude and adjusted effect | | 100.0% | 1.00 | 88.9% | 0.78 | 88.9% | 0.78 | 89% |
| 5.More than one adjusted model | | 100.0% | 1.00 | 88.9% | 0.73 | 88.9% | 0.73 | 89% |
| Number of items | |  |  |  |  |  |  |  |
|  | Intraclass correlation coefficient: | 0.964 | | 0.860 | | 0.899 | | 0.885 |

TABLE S2 (b). Intra-rater Agreement on Each Item: Comparison of Percentage of Agreement

|  |  | Reviewer 1 | | Reviewer 2 | | Reviewer 3 | |
| --- | --- | --- | --- | --- | --- | --- | --- |
| Item description |  | n=136 | | n=140 | | n=143 | |
|  | *P value^a^* | n | % | n | % | n | % |
| 1. Model assumptions and goodness-of-fit | 0.530 | 33 | 24.3% | 34 | 24.3% | 42 | 29.4% |
| 2. Interaction analysis | 0.095 | 33 | 24.3% | 21 | 15.0% | 23 | 16.1% |
| 3. Sensitivity analysis | 0.148 | 46 | 33.8% | 37 | 26.4% | 53 | 37.1% |
| 4. Crude and adjusted effect | 0.343 | 52 | 38.2% | 46 | 32.9% | 43 | 30.1% |
| 5. More than one adjusted model | 0.984 | 35 | 25.7% | 35 | 25.0% | 37 | 25.9% |
| a= p value computed with chi square test. |  |  |  |  |  |  |  |
